# Sex and age differences in patients treated surgically for acute subdural hematoma

**DOI:** 10.1016/j.bas.2025.105602

**Published:** 2025-09-06

**Authors:** Anna Koller, Christoph Reich, Claudius Thomé, Daniel Pinggera

**Affiliations:** a Department of Neurosurgery, Medical University of Innsbruck, Innsbruck, Austria; b Department of Internal Medicine III, Cardiology, University Hospital of Heidelberg, Heidelberg, Germany

**Keywords:** Acute subdural hematoma, Sex differences, Age-related differences, Decompressive craniectomy, Craniotomy, Traumatic brain injury

## Abstract

**Introduction:**

Acute subdural hematoma (aSDH) is a severe condition with high mortality despite advances in neurosurgical care. Predicting outcomes remains challenging due to individual variability. This study explores sex- and age-related differences in surgical treatment and outcomes of aSDH.

**Research question:**

How does sex and age influence surgical decision-making and outcomes in patients undergoing surgery for acute subdural hematoma?

**Material and methods:**

We retrospectively analyzed 328 patients treated surgically for aSDH between 2005 and 2015. Demographic data, clinical characteristics (GCS), radiological parameters, surgical approach (osteoplastic craniotomy [OC] vs. decompressive craniectomy [DC]), and outcomes (Glasgow Outcome Scale [GOS]) at discharge were collected.

**Results:**

The cohort included 211 men (mean age: 56) and 117 women (mean age: 68). Hematoma volumes were similar between sexes (p = 0.9), yet surgical choices differed significantly (OC in 58 % of women vs. 43 % of men; p = 0.008). Elderly patients (>70 years; n = 133) had larger hematoma volumes than younger patients (64.6 vs. 44.8 cm^3^; p < 0.0001) and were more frequently treated with OC (68 % vs. 35 %; p < 0.0001). Age and hematoma volume—but not sex—were independent predictors of surgical approach. Outcome (GOS) was worse in elderly patients (p < 0.001) and those undergoing DC (p = 0.001). Time to CT correlated with outcome (p = 0.001), while time to surgery did not.

**Discussion and conclusion:**

Despite comparable hematoma volumes, surgical strategies varied by sex and age. Elderly patients with larger hematomas were less likely to receive DC. These findings highlight demographic influences on surgical decision-making and support a more individualized approach in managing aSDH.

## Highlights

• Elderly patients with aSDH had larger hematoma volumes than younger patients.
• Women were more often treated with craniotomy, men more with decompressive craniectomy.
• Despite larger hematomas, elderly patients less frequently underwent craniectomy.
• Hematoma volume and age, but not gender, predicted surgical treatment type.
• Outcome was worse in older patients and those undergoing decompressive craniectomy.

## 1 Introduction

Traumatic Brain Injury (TBI) is a significant public health and socioeconomic issue, standing as the primary cause of mortality and morbidity among young adults (Wilson et al., 2014). It impacts approximately 1.7 million individuals annually in the United States, resulting in 52,000 deaths and over 275,000 hospitalizations (Faul et al.). In Europe, the incidence reaches 262 per 100,000 patients yearly (Peeters et al., 2015). TBI exhibits a sex discrepancy, being more prevalent among males, with a male-to-female ratio ranging from 1.2:1.0 to 4.6:1.0 (Peeters et al., 2015; Wilberger et al., 1991; Karibe et al., 2014). However, recent observations have noted changes in TBI epidemiology over the past decade, with an increasing incidence among elderly individuals, particularly elderly women (Peeters et al., 2015; Petridis et al., 2009). Ten to twenty percent of TBI patients present with an acute subdural hematoma (aSDH). Among severe TBI cases, defined by a Glasgow Coma Scale (GCS) score of 8 or less, the prevalence of aSDH rises to 60 % (Wilberger et al., 1991; Karibe et al., 2014). Mortality rates from aSDH remain high, ranging from 40 to 60 %, with only 19–45 % achieving functional recovery (Li et al., 2012). Additionally, mortality escalates to about 70 % if the GCS score on admission is 8 or less (Karibe et al., 2014). Despite various treatment options, morbidity and mortality rates remain elevated (Bullock et al., 2006). Surgical evacuation techniques vary, including simple burr hole trephination, osteoplastic craniotomy (OC), and decompressive craniectomy (DC), with OC and DC being the most common. However, the superiority of either procedure has yet to be established (Li et al., 2012; Chen et al., 2011; Kwon et al., 2016; Rush et al., 2016; Van Essen et al., 2017; Hutchinson et al., 2023). Studies consistently associate age over 65 years with poor outcomes and mortality rates notably increase in patients over 70 years old (Mauritz et al., 2014; Leitgeb et al., 2012) with a GCS score of 9 or less (Peeters et al., 2015; Wilberger et al., 1991; Leitgeb et al., 2012; Jamjoom, 1992; Cagetti et al., 1992).

The impact of sex on outcome however remains unclear (Farace and Alves, 2000; Marhold et al., 2022, Munivenkatappa et al., 2016). While the overall incidence of TBI is up to four times higher in men than in women, women appear to be equally affected during childhood and after the age of 45 (Peeters et al., 2015; Henry et al., 1992; Pentland et al., 1986). Farace and Alves' meta-analysis in 2000 indicated worse outcomes for women in 85 % of measured variables, including mortality and hospitalization duration (Farace and Alves, 2000). However, Leitgeb et al. reported that female sex is not an independent risk factor for in-hospital mortality (Leitgeb et al., 2011).

The goal of this study was to identify sex and age disparities in surgically treated aSDH by examining demographic, clinical factors, and radiological measurements. Since most aSDH patients are initially diagnosed using a native CT scan, we chose for volumetric measurements due to their reliability, reproducibility, and decreased susceptibility to tilt affections.

## 2 Material and Methods

### 2.1 Study design and overview

All patients who underwent surgical treatment for aSDH at our department between 01/2005 and 12/2015 were retrospectively reviewed. Our primary interest focused on patients treated with hematoma evacuation, wherefore patients with only probe placement, non-traumatic aSDH, subacute/chronic subdural hematoma or unavailable medical records were excluded. Out of a total of 343 patients presenting with aSDH, 328 patients met the inclusion criteria and were thus included in the study. Treatment of all patients adhered to the principles outlined in the Declaration of Helsinki and complied with Austrian regulations. The study protocol was approved by the Medical University Innsbruck's ethics committee (Protocol number: AN 1393/2022). Demographic and clinical data were gathered from medical records, including patient age, sex, initial GCS score (first documented GCS), surgical procedure (OC or DC), time interval from trauma to CT scan and time interval from trauma to surgical procedure. The choice of surgical procedure and the indication for surgery were determined by the treating specialist, based on current literature and individual patient factors. A standardized protocol was not applied. TBI severity was classified based on the GCS score within 48 h into severe (GCS = 3–8), moderate (GCS = 9–12), and mild (GCS = 13–15) TBI. Regarding age, the study population was categorized into two groups: 1. an elderly group defined as age over 70 years, and 2. a younger group defined as age less than 70 years. The primary outcome measure was the GOS (rating from 1 to 5) at discharge. GOS outcomes were dichotomized into unfavorable outcome (GOS 1 to 3) and favorable outcome (GOS 4 to 5) at discharge. A later endpoint was not feasible as due to the touristic orientation in our region, many foreign patients are treated at our center and after acute management, these patients are typically transported back to their home countries, resulting in a loss of follow-up. Imaging data were analyzed using Icoserve Advanced Imaging Management (V.1.8.8.34, ITH Icoserve Technology for Healthcare GmbH, Innsbruck, Austria) and iPlanNet (V. 3.1, BrainLab Inc., Feldkirchen, Germany). Three-dimensional volumetry of aSDH using manual segmentation was conducted on the first available non-enhanced CT scan after injury and before treatment (consisting of 27–65 slices). The Hematoma was manually delineated in all relevant slices. iPlanNet software computed volumes based on manually selected areas, employing interpolation to segment pixels according to radiodensity properties measured in Hounsfield Units (HU). Radiological assessments, including 3D-volumetry, were performed from one person of the institute and subsequently reviewed by another neurosurgeon who was blinded to the patient's clinical course.

### 2.2 Statistical analysis

Statistical analyses were conducted using IBM SPSS Statistics (V.24, version 24, SPSS Inc., IBM, Chicago, IL, USA). Differences with a p-value of less than 0.05 were considered statistically significant. In the univariate analysis, group differences were determined using the chi-square test for dichotomized variables. Continuous variables were analyzed using the independent samples *t*-test and the Mann-Whitney *U* test, as appropriate. Spearman correlation analysis was used to evaluate the effect of the different volumes on patient outcome. To identify independent predictors of the type of surgery (osteoplastic vs. osteoclastic), a multivariate analysis was performed using binary logistic regression including age, sex, and hematoma volume as covariates.

## 3 Results

### 3.1 Demographic and clinical data

This retrospective study analyzed 328 patients with aSDH over a 10-year period. There were 211 (64.3 %) male and 117 (35.7 %) female participants with a mean age of 56 years and 68 years, respectively. The age differed significantly between men and women (p < 0.001). The average GCS of the 211 men was 10, with 43 % having severe traumatic brain injuries, 10 % having moderate injuries, and 47 % having mild injuries. In the 117 women, the mean GCS was 11. Fifty-five percent of these had severe traumatic brain injury, 14 % had moderate traumatic brain injury and 31 % had mild traumatic brain injury. The initial GCS showed no statistical difference between men and women. Mean GOS at discharge was 3.0 in men and 2.7 in women which showed no statistical significance in that term outcome (p = 0.117). [Table 1].

**Table 1:** Sex-specific demographics.

	Men	Women	P value
Number	211	117	
Age (mean)	56	68	<0.001
GCS (mean)	10	11	0.075
Percentage mild (GCS 13–15)	47	31	
Percentage moderate (GCS 9–12)	10	14	
Percentage severe (GCS 3–8)	43	55	
GOS (mean)	3.0	2.7	0.117

### 3.2 Imaging data

Mean hematoma volume was 52.9 cm^3^ in men and 52.4 cm^3^ in women. There were no significant differences in the mean hematoma volume between both sex groups (p = 0.9). Larger hematoma volume was only significantly related to worse outcome measured on dichotomous GOS at discharge in men (p < 0.001), but not in women (p = 0.096).

The time from trauma to the first CT scan amounted to an average of 7.5 h for men, while an average of 9.4 h was recorded for women. This showed a difference with a statistical

significance of p = 0.026. Mean time from trauma to surgical intervention was 15.4 h for men and 18.0 h for women. This also showed a statistical significance (p = 0.016).

Out of 211 male patients, OC was performed in 90 (43 %) patients and DC was performed in 121 (57 %) patients, whereas OC was performed in 68 (58 %) female patients and DC was performed in 49 (42 %) female patients (p = 0.008). There were no significant differences in the neurological outcome between both groups (p = 0.860). [Table 2].

**Table 2:** Sex-specific radiological and surgical data.

	Men	Women	P value
Volume hematoma(cm^3^)	52.9	52.4	0.883
Maximal Hematoma depth (mm)	14.0	14.0	0.700
Midline-Shift (mm)	9.5	8.8	0.998
Time accident- surgery (h)	15.4h	18.0h	0.016
DC:OC	121:90	49:68	0.008

### 3.3 Age

When corrected for age, we could not find differences between the sexes anymore, but did find differences between age groups: age, initial GCS, hematoma volume, ratio DC:OC, and outcome measured by GOS score. Out of all 328 patients, 133 patients belonged to the elderly group (>70 years, 40.6 %, m:f = 1:1) and 195 patients belonged to the younger group (<70 years, 59.4 %, m:f = 3:1). In the elderly, men had an average age of 79 years and women of 81 years, with both sexes having a median initial GCS of 11. The mean hematoma volume was 70 cm^3^ for men and 60 cm^3^ for women. Operative treatment was performed with a craniectomy to craniotomy ratio of 22:41 for men and 21:49 for women. The GOS was 2.4 for men and 2.4 for women. In the younger group, men had an average age of 47 and women 49, with both sexes having a median initial GCS of 9. The mean hematoma volume was 45.8 for men and 49 for women. Operative treatment was performed with a craniectomy to craniotomy ratio of 99:49 for men and 28:19 for women. The GOS was 3.2 for men and 3.0 for women. The data are listed in Table 3 [Table 3]*.*

**Table 3:** Radiological, surgical and outcome data regarding age groups.

Characteristics	Age group∗	Men	Women	P value
Number	>70 years	63	70	
	<70 years	148	47	
Age (mean, years)	>70 years	79	81	p = 0.062
	<70 years	47	49	p = 0.249
Initial GCS(3–15, mean)	>70 years	11	11	p = 0.588
	<70 years	9	9	p = 0.435
Hematoma volume (cm^3^, mean)	>70 years	70.0	60.0	p = 0.213
	<70 years	45.8	40.8	p = 0.390
Maximal Hematoma depth (mm)	>70 years	16.4	15.4	p = 0.493
	<70 years	13.0	12.0	p = 0.522
Midline-Shift (mm, mean)	>70 years	9.2	9.1	p = 0.772
	<70 years	9.7	8.5	p = 0.603
Time injury- surgery (h)	>70 years	11.6	8.9	p = 0.924
	<70 years	6.3	8.3	p = 0.656
DC (n): OC (n)	>70 years	22:41	21:49	p = 0.828
	<70 years	99:49	28:19	p = 0.640
GOS (1–5, meaan)	>70 years	2.5	2.4	p = 0.433
	<70 years	3.2	3.0	p = 0.427
Favorable GOS (4–5, mean)	>70 years	43 %	36 %	p = 0.261
	<70 years	38 %	42 %	p = 0.296

In the multivariate logistic regression analysis evaluating predictors of surgical technique (OC: DS), age at surgery and hematoma volume were independently associated with the type of procedure performed. Increasing age was significantly associated with a decreased likelihood of undergoing osteoclastic surgery (Exp(B) = 0.960, p < 0.001), indicating that each additional year of age reduced the odds of osteoclastic surgery by approximately 4 %. Hematoma volume showed a borderline significant association (Exp(B) = 1.006, p = 0.050), suggesting that larger hematoma volumes slightly increased the likelihood of osteoclastic intervention. Sex was not a significant predictor of surgical type (Exp(B) = 0.886, p = 0.675).

### 3.4 Outcome

Overall, 196 (59.8 %) patients (120 men, 76 women) showed an unfavorable outcome and 132 (40.2 %) (91 men, 41 women) a favorable outcome measured on dichotomous GOS at discharge. Overall, the mean GOS at discharge was 2.9, with men averaging 3.0 and women averaging 2.7 (p = 0.117).

Age at surgery was significantly associated with outcome regarding GOS both in male (p < 0.001) and female (p = 0.006) with worse outcome in the elderly. Although men had better outcomes, there was no significant difference in the Glasgow Outcome Scale (GOS) at discharge between male and female patients (3.0:2.7). Furthermore, there was both no significant difference between sexes measured on dichotomous GOS at discharge (p = 0.140), and regarding age groups (the younger group p = 0.485, the elderly group p = 0.404).

The mean initial GCS was 10 in men and 11 in women. Initial GCS difference was not significant between the men and the women (p = 0.075). In both groups, initial GCS correlated significantly with outcome (male p < 0.001, female p = 0.001).

Larger hematoma volume in men and women was both significantly associated with a worse GOS at discharge (p = < 0.001).

We also performed a multivariate analysis for outcome, including sex, hematoma volume, age at surgery, and initial GCS as predictors. Neither sex (OR 1.427, p = 0.174) nor hematoma volume (OR 0.999, p = 0.867) were significantly associated with outcome. In contrast, higher age at surgery was linked to worse outcome (OR 0.981, p = 0.005, 95 % CI 0.968–0.994), while higher initial GCS predicted better outcome (OR 1.048, p = 0.027, 95 % CI 1.006–1.093).

Number of performed DCs and OCs were nearly similar: 170 patients (51.8 %) underwent DC and 158 (48.2 %) OC. Mean age at surgery was 56 years in men and 68 years in women (p < 0.001). The group undergoing DC was significantly younger (mean age in DC 54 years, and in OC 68 years, p < 0.001). Initial GCS was significantly less in the DC group (mean initial GCS 8 in DC, and 12 in OC, p < 0.001). We found no significant difference in hematoma volume between the two surgical groups (mean hematoma volume in DC 52.6 cm^3^, and in OC 52.8 cm^3^, p = 0.964). Yet there were significant differences in volumes of the lateral (mean volume of lateral ventricle in DC 13.3 cm^3^, and in OC 20.2 cm^3^, p < 0.001) and the third ventricle (mean volume of the third ventricle in DC 0.7 cm^3^, and in OC 1.1 cm^3^, p = 0.009).

Outcome measured on GOS at discharge was significantly worse in the DC group (mean GOS at discharge in DC 2.6, and in OC 3.1, p = 0.001).

Our data showed a significant correlation between time from injury to first CT scan and outcome (n = 284, mean time from injury to first CT scan 5.2 h in the worse outcome group vs 12.3 h in the favorable outcome group, p = 0.001). Correlation between time of injury to surgery and outcome was not significant (n = 177, time from injury to surgery 12.0 h in the worse outcome group vs 20.7 h in the favorable outcome group, p = 0.051). However, there was an apparent trend showing the longer time of injury to surgery, the better the outcome (see Table 4).

**Table 4:** Overall Comparison of GOS outcomes by sex (GOS good = GOS 4–5, GOS bad = GOS 1–3).

Variable	Total Count	GOS good (♂)	GOS good (♀)	GOS bad (♂)	GOS bad (♀)	p-value
Count	328	91	41	120	76	
Age (years)		49.5	61.2	62.1	72.1	0.04
GCS (Glasgow Coma Scale)		11.3	13.0	8.3	9.5	0.001
Hematoma Volume (mm^3^)		39.0	44.3	63.9	57.5	0.004
Craniectomy: Craniotomy		45:4	37:3	76:44	12:2	0.03
		6	9		9	

## 4 Discussion

Despite the higher overall incidence of TBI in men, the influence of sex on outcomes in the elderly population remains insufficiently explored in the literature. Interestingly, while men typically undergo more aggressive surgical techniques for hematoma evacuation than women, our data revealed no significant differences in neurological outcomes between sexes (Leitgeb et al., 2012). Previous studies, such in a meta-analysis suggested that women fare worse across various outcomes, including higher mortality rates and longer hospital stays (Farace and Alves, 2000).

In contrast, our findings align more closely with those of another study, who concluded that female sex is not an independent risk factor for in-hospital mortality (Leitgeb et al., 2012). An analysis of the National Trauma Data Bank registry suggested that sex had minimal impact on overall in-hospital mortality. However, it also highlighted that older women were more frequently affected by aSDH (Kerezoudis et al., 2020). Accordingly, a review examining multiple outcome parameters did not identify a significant correlation between sex and clinical outcomes (Manivannan et al., 2021). Comparable findings were observed in the study by Shibahashi et al., where sex was not identified as a negative prognostic factor. However, unlike our study, their age cut-off was set at 60 years, and their analysis primarily focused on in-hospital mortality (Shibahashi et al., 2019).

Rapid transfer to a neurosurgical hospital and early surgical intervention of an aSDH has historically been linked to better outcomes (Bullock et al., 2006; Seelig et al., 1981). There was reported significantly lower mortality (30 %) and better functional recovery (65 %) in patients operated within 4 h of injury, compared to 85 % mortality and 7 % recovery in those treated later (Seelig et al., 1981). Similar data have described a significant impact of time from injury to surgery on functional recovery. However, these findings should be interpreted with caution, as they are based on a very small cohort (Chen et al., 2020). In contrast, our study found no significant correlation between injury-to-surgery time and patient outcomes, which aligns with the findings of Fountain et al., who reported similar results based on a 20-year study in the UK (Fountain et al., 2017). Additionally, other studies on aSDH management have found limited evidence supporting a direct link between time to surgery and clinical outcomes. (Wilberger et al., 1991; Karibe et al., 2014; Servadei et al., 2000). They emphasized that the interval between clinical deterioration and surgery might be more critical. Another study showed that patients operated within 2 h of clinical decline had better outcomes than those treated later (Haselsberger et al., 1988). Notably, patients undergoing early surgery often had more severe injuries, complicating direct comparisons between groups (Potts et al., 2012). Interestingly, our data show that women experience a longer interval between injury and surgery than men, but this did not impact outcomes. Timing may not be as critical as previously thought, with other factors, such as the time between clinical deterioration and surgery. This is supported by a large multicenter cohort of patients with aSDH, where early surgical intervention was not associated with improved functional outcomes. Similarly, Trevisi et al. demonstrated that, after adjusting for initial clinical status, surgical timing itself did not independently predict survival or outcome, with radiological factors such as midline shift being more decisive (31,32).

Regarding surgical interventions for aSDH we observed significant differences in outcomes between decompressive craniectomy (DC) and craniotomy (OC). DC was associated with a higher complication rate and poorer outcomes, and patients undergoing DC presented with a worse preoperative clinical status. This likely reflects a selection bias, where surgeons choose DC for more severe cases, leading to worse outcomes (Chen et al., 2011). Age also influenced surgical decision-making, with OC being more commonly performed in older female patients. Another study provided insights into surgical outcomes for aSDH by comparing decompressive craniectomy (DC) and craniotomy (OC) (Kerezoudis et al., 2020). Their study highlighted a higher inpatient mortality rate associated with decompressive craniectomy (DC), even after adjusting for baseline clinical severity. Additionally, they found that DC was more frequently performed in younger male patients with severe injuries, reinforcing our observations on the influence of demographic and clinical factors on surgical decision-making (Kerezoudis et al., 2020). Similar trends have been reported in other studies, where men and younger patients were more likely to undergo DC, which was also associated with worse outcomes (van Essen et al., 2022; Trevisi et al., 2022). A key study in this context is the RESCUE-ASDH trial by Hutchinson et al., which compared decompressive craniectomy with craniotomy in ASDH and found no significant difference in long-term functional outcome, though craniectomy carried more complications and reoperations. However, direct comparison to our series is difficult, as RESCUE-ASDH always employed a large bone flap and focused only on whether it was replaced or left out, whereas our analysis also considered different craniotomy sizes in addition to decompressive procedures (Hutchinson et al., 2023).

Additionally, we observed a higher incidence of aSDH in elderly women, aligning with findings that TBI prevalence is highest among individuals under 25 and over 75 years, with distinct injury mechanisms across age groups (Shibahashi et al., 2019). Contrary, a higher proportion of men suffered severe TBI, whereas no significant differences were observed in key parameters within the same age group, suggesting that age may be a more influential factor than sex in shaping treatment decisions and outcomes. Recent findings from the literature provide additional context to our observations. One study reported that female TBI patients, particularly in pediatric and elderly populations, exhibited higher mortality rates and more severe injuries than their male counterparts (Chen et al., 2017). These findings contrast with our results, which showed no significant sex differences in outcomes. However, the demographic-specific variations in injury severity observed in that study are consistent with our data, reinforcing the notion that sex alone may not be a reliable predictor of clinical outcomes. Additionally, another study introduced a stratification framework aimed at refining the classification of aSDH patients. The study found that high-velocity impact (HVI) injuries were associated with significantly worse outcomes, including higher mortality rates and prolonged hospital stays (Phan et al., 2017). These findings align with our results, highlighting the critical role of injury severity and mechanism in determining patient outcomes. Implementing such stratification systems could enhance data analysis and improve consistency in future research.

Hematoma volume and depth did not differ significantly between sexes, though a larger hematoma volume was associated with worse outcomes. This is consistent with previous studies indicating that greater hematoma volume is a negative prognostic factor (Kerezoudis et al., 2020). Further analysis of radiological parameters supported this observation, demonstrating that larger hematoma volumes are linked to poorer clinical outcomes (Manivannan et al., 2021; Shibahashi et al., 2019).

## 5 Limitations

Our study has several limitations. First, data collection and statistical analysis were performed retrospectively. Patient outcomes were only assessed at discharge rather than at defined follow-up intervals. Furthermore, because our department is located in a high-volume tourist region, many international patients with major trauma are referred for acute management but often return to their home countries thereafter, resulting in loss to follow-up. In addition, we only analyzed surgically treated cases of acute subdural hematoma, which may not fully represent the entire patient population. Another limitation is that treatment decisions were made at the discretion of the attending specialist without a standardized protocol, which may have introduced variability in surgical indications and procedures. Nevertheless, given the size of our center, the relative uniformity of the treatment approach, and the large number of patients included, we believe that individual nuances were minimized and the impact of these limitations attenuated.”

## 6 Conclusion

This study examined sex and age-specific differences in treatment outcomes for patients with aSDH. Results indicate that women are more frequently treated osteoplastically and experience longer times to CT scans or surgeries. However, no sex-specific differences in outcomes were found within age groups. Men had higher hematoma volumes, correlating with poorer outcomes. These findings highlight the importance of considering sex-specific factors in evaluating and treating acute subdural hematomas. An individualized approach based on sex could enhance diagnostic procedures and neurosurgical treatment strategies, potentially improving health outcomes and postoperative care for traumatic brain injuries.

## Ethics approval

Approved by the Ethics Committee of the Medical University of Innsbruck (AN 1393/2022).

## Funding

No specific funding was received for this study.

## Conflict of interest

The authors declare no competing interests.
